# EZH2 is required for parathyroid and thymic development through differentiation of the third pharyngeal pouch endoderm

**DOI:** 10.1242/dmm.046789

**Published:** 2021-03-12

**Authors:** Cinzia Caprio, Gabriella Lania, Marchesa Bilio, Rosa Ferrentino, Li Chen, Antonio Baldini

**Affiliations:** 1 Institute of Genetics and Biophysics, Consiglio Nazionale delle Ricerche, 80131 Naples, Italy; 2 Department of Biology and Biochemistry, University of Houston, Houston TX 77204, USA; 3 Department of Molecular Medicine and Medical Biotechnologies, University Federico II, 80131 Naples, Italy

**Keywords:** Ezh2, Parathyroids, Pharyngeal endoderm, Tbx1

## Abstract

The *Ezh2* gene encodes a histone methyltransferase of the polycomb repressive complex 2 that methylates histone H3 lysine 27. In this study, we investigated whether EZH2 has a role in the development of the pharyngeal apparatus and whether it regulates the expression of the *Tbx1* gene, which encodes a key transcription factor required in pharyngeal development. To these ends, we performed genetic *in vivo* experiments with mouse embryos and used mouse embryonic stem cell (ESC)-based protocols to probe endoderm and cardiogenic mesoderm differentiation. Results showed that EZH2 occupies the *Tbx1* gene locus in mouse embryos, and that suppression of EZH2 was associated with reduced expression of *Tbx1* in differentiated mouse ESCs. Conditional deletion of *Ezh2* in the *Tbx1* expression domain, which includes the pharyngeal endoderm, did not cause cardiac defects but revealed that the gene has an important role in the morphogenesis of the third pharyngeal pouch (PP). We found that in conditionally deleted embryos the third PP was hypoplastic, had reduced expression of *Tbx1*, lacked the expression of *Gcm2*, a gene that marks the parathyroid domain, but expressed *FoxN1*, a gene marking the thymic domain. Consistently, the parathyroids did not develop, and the thymus was hypoplastic. Thus, *Ezh2* is required for parathyroid and thymic development, probably through a function in the pouch endoderm. This discovery also provides a novel interpretational key for the finding of *Ezh2* activating mutations in hyperparathyroidism and parathyroid cancer.

## Abstract (precis)

**Summary:** EZH2 in the pharyngeal apparatus is required in the endoderm for the development of the parathyroids and thymus, and positively regulates the expression of the *Tbx1* gene.

## INTRODUCTION

The polycomb chromatin remodelling complex (PcG) is a key transcriptional regulator that represses gene expression. EZH2 is part of the polycomb repressive complex 2 (PRC2) and has methyltransferase activity that methylates lysine 27 of histone H3 to generate H3K27me3. Although there is a similar gene in the mouse genome, named *Ezh1* (Shen et al., 2008), *Ezh2* is indispensable and its loss causes embryonic lethality at or before gastrulation (O'Carroll et al., 2001). However, in some cases functional redundancy has been noted (Ezhkova et al., 2011; Shen et al., 2008). *Ezh2* is required for a number of developmental processes (Aloia et al., 2013). Specifically, several reports have addressed the role of *Ezh2* during heart development using tissue-specific deletion of the gene. Ablation in the *Nkx2-5*^Cre^ recombination domain, which encompasses the first and second heart fields (SHF) as well as the pharyngeal endoderm, resulted in cardiac defects (Chen et al., 2012; He et al., 2012). However, ablation using the Mef2c-AHF-Cre driver, which induces recombination in the SHF and right ventricle, resulted in postnatal cardiomyocyte hypertrophy, but no morphogenetic defects of the outflow tract (Delgado-Olguín et al., 2012), suggesting that *Ezh2* is dispensable during SHF development. Nevertheless, *Ezh2* was shown to de-repress *TBX1* expression in human embryonic stem cells (ESCs) (Collinson et al., 2016). In addition, it has been shown that inactivation of the *Eed* gene, which encodes another component of the PRC2 complex, in the *FoxN1* domain of the endodermic thymic primordia is associated with upregulation of *Tbx1* gene expression in the developed thymus (Singarapu et al., 2018). These findings suggest that *Tbx1* may be a target of PRC2. *Tbx1* is an important player in the development of the SHF, cardiopharyngeal mesoderm and the pharyngeal endoderm. The gene is strongly implicated in DiGeorge/22q11.2 deletion syndrome, a developmental disorder that affects the pharyngeal apparatus (Baldini et al., 2017).

Here, we have addressed the question of whether *Tbx1* is a target of EZH2. To this end, we have used genetically modified mouse lines and differentiating mouse ESCs. Results showed that the EZH2 protein binds to the *Tbx1* gene and affects its expression in a tissue-specific manner. However, in contrast with the canonical function of EZH2, its loss is associated with reduced expression of the *Tbx1* gene. Conditional deletion in the *Tbx1* expression domain showed that *Ezh2* is a modifier of the *Tbx1* mutant phenotype and is required for parathyroid and thymic development.

## RESULTS

### EZH2 localizes to the *Tbx1* gene in mouse embryos

**Fig. 1.**
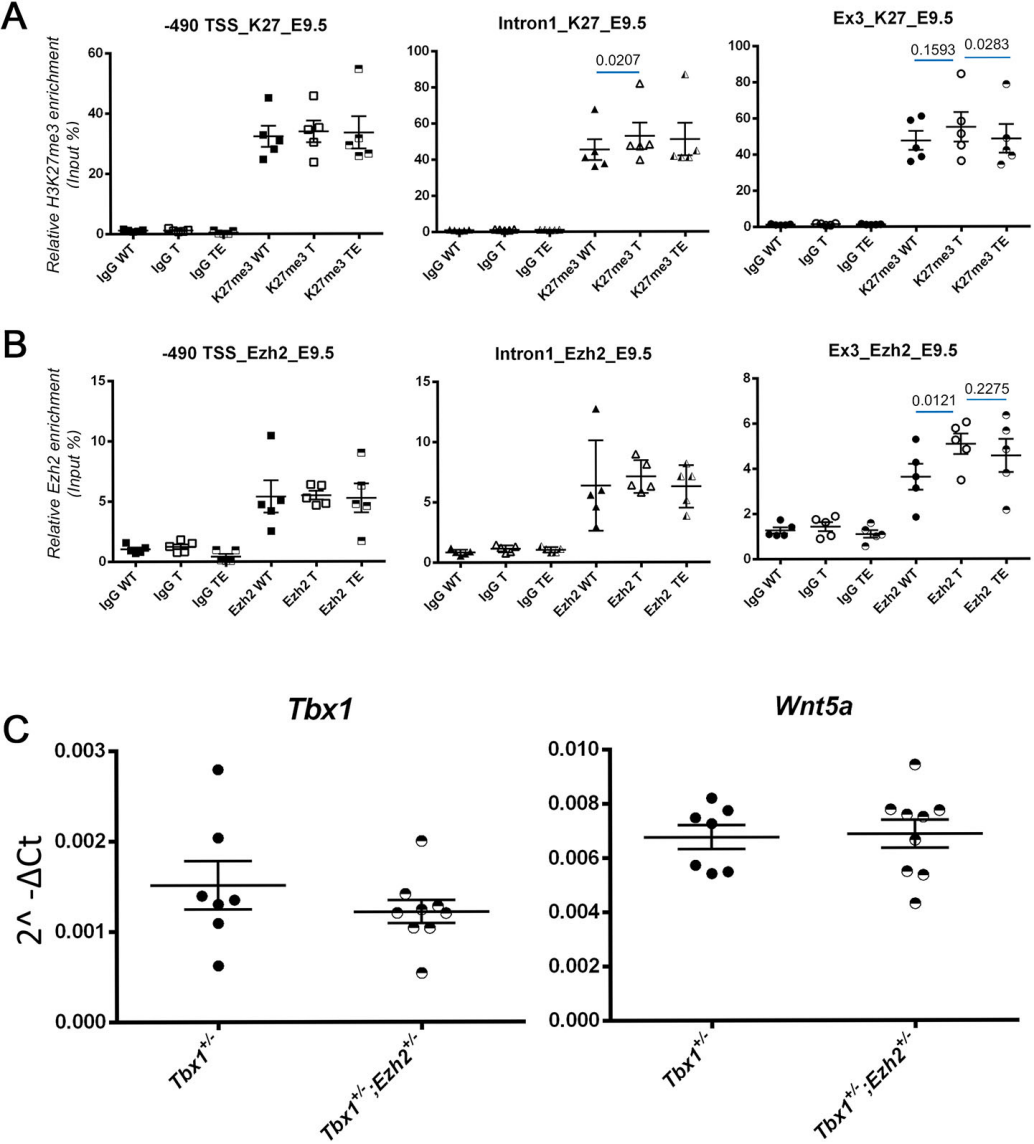
**H3K27me3 and EZH2 enrichment at the *Tbx1* locus, and gene expression.** (A) qChIP experiments using an anti H3K27me3 antibody on three loci of the *Tbx1* gene on chromatin from whole E9.5 mouse embryos, wild type (WT), *Tbx1*^+/−^ (T), and *Tbx1*^+/−^;*Ezh2*^+/−^ (TE). Significant differences in H3K27me3 were detected at intron 1 (increased in T) and exon 3 loci (reduced in TE compared to T). (B) EZH2 qChIP using E9.5 mouse embryos. Note a significant increase of EZH2 enrichment at the Exon 3 locus of *Tbx1*^+/−^ embryos. Each dot represents an independent experiment using one embryo. (C) Gene expression measured by quantitative real time PCR of cDNA from whole E8.5 embryos (*Wnt5a* is a transcriptional target of TBX1). We found no change of expression of the two genes in *Tbx1*^+/−^;*Ezh2*^+/−^ embryos compared to *Tbx1*^+/−^ embryos. Each dot represents a single embryo. Data are mean±s.e.m. Figures above the blue segments indicate *P* values, calculated by a paired two-tailed Student's *t*-test using GraphPad software.

Published data sets show that the mouse *Tbx1* gene region is enriched for H3K27me3 in various stages of mouse ESC differentiation (Wamstad et al., 2012) (Fig. S1A). To establish whether H3K27me3 enrichment also occurs *in vivo*, we performed quantitative chromatin immunoprecipitation (qChIP) of embryonic day (E)9.5 mouse embryos and tested H3K27me3 enrichment at three loci. We found high enrichment at all the loci tested (Fig. 1A). The enrichment of H3K27me3 increased in *Tbx1*^+/−^ embryos relative to wild-type embryos in intron 1, but it did not change in the other two loci tested. In *Tbx1*^Cre/+^;*Ezh2*^flox/+^ embryos, we found a modest reduction of H3K27me3 enrichment compared to *Tbx1^Cre^*^/+^ embryos (heterozygous mutants) but only in the exon 3 locus (Fig. 1A). Next, we performed qChIP using an anti-EZH2 antibody and found localization of the EZH2 protein at *Tbx1* gene loci. We detected an effect of the genotype only at the exon 3 locus in *Tbx1^cre^*^/+^ embryos, in which we found a slight increase in EZH2 enrichment compared to wild-type embryos (Fig. 1B).

We next tested the expression of *Tbx1* and *Wnt5a*, a *Tbx1* target, by qRT-PCR on RNA from somite-matched whole E8.5 embryos with the genotypes *Tbx1*^+/−^;*Ezh2*^+/+^ versus *Tbx1*^+/−^;*Ezh2*^+/−^. Results showed that *Ezh2* heterozygosity had no significant effect on *Tbx1* or *Wnt5a* expression (Fig. 1C).

The lack of significant expression changes of *Tbx1* in *Ezh2* heterozygous mutant embryos may be because of the heterogeneity of the tissue tested (whole embryo) or because the heterozygous deletion is insufficient to significantly reduce the availability of the EZH2 protein to the chromatin. Therefore, we switched to a mouse ESC-based system to study differentiated cell types.

### Loss of EZH2 or inhibition of its enzymatic activity during differentiation of ESCs downregulates *Tbx1* gene expression

**Fig. 2.**
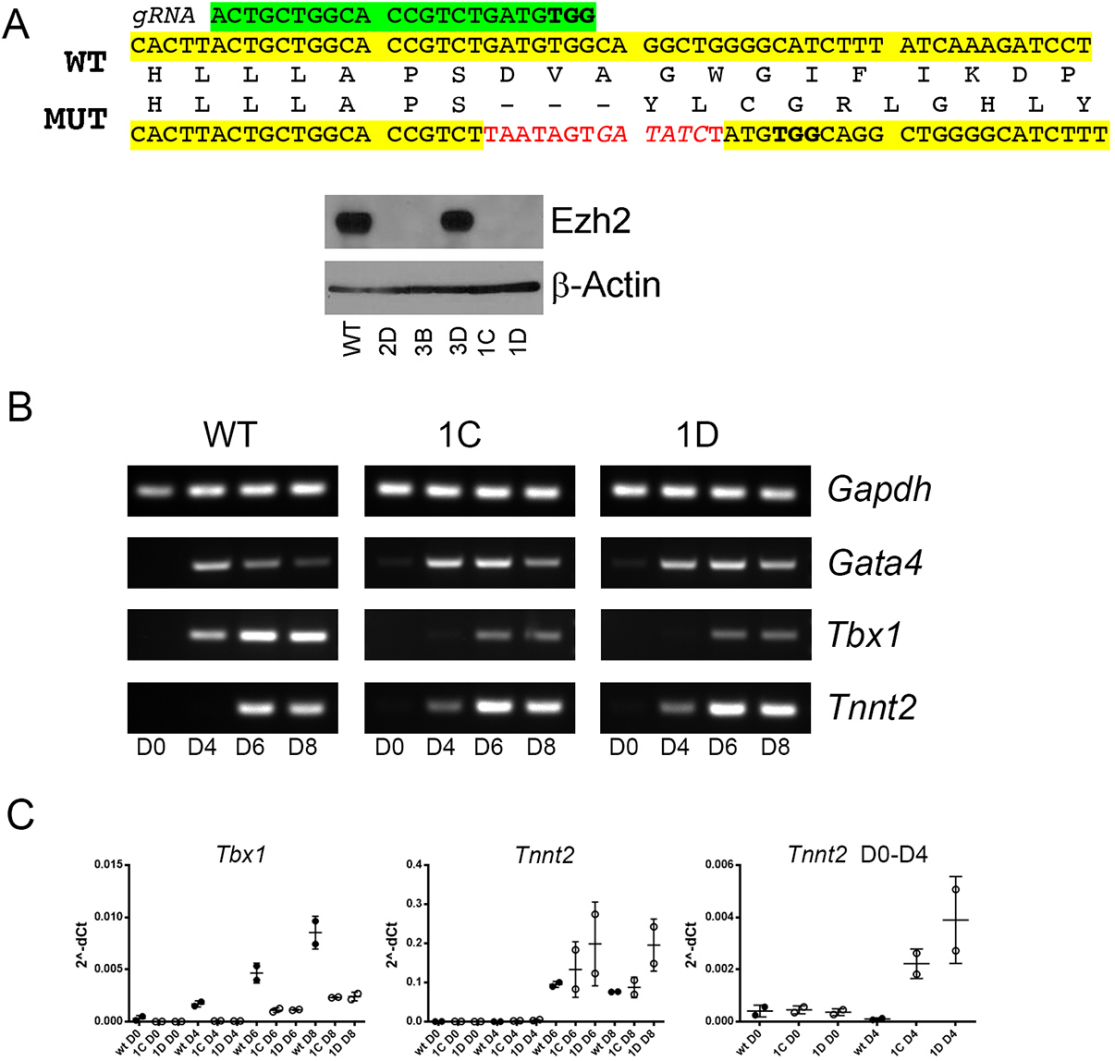
***Ezh2*^−/−^ mouse ESCs differentiate into cardiac mesoderm but express lower levels of *Tbx1*.** (A) Generation of *Ezh2*^−/−^ mouse ESC clones using CRISPR/Cas9. In green is the gRNA sequence and protospacer adjacent motif (bold) sequence. In yellow is the wild-type (WT) sequence of exon 16, and red indicates the sequence inserted by homologous recombination, confirmed for clones 1C and 1D. The inserted sequence includes multiple stop codons and causes a downstream frameshift. The western blot with an anti EZH2 antibody shows four clones with no detectable signal. Clones 1C and 1D were used for subsequent experiments. MUT, mutant. (B,C) Cardiac mesoderm differentiation using a previously reported protocol (Andersen et al., 2018). (B) *Tbx1* expression is clearly detectable at day 4 of differentiation, and is stronger at day 6 and day 8 in wild-type cells. In *Ezh2*^−/−^ cells, *Tbx1* was only weakly expressed at day 6 and 8, whereas the expression of differentiation marker cardiac troponin (*Tnnt2*) appeared earlier than in wild-type cells, suggesting premature differentiation. These observations were confirmed by quantitative real time PCR (C) in two independent experiments. Data are mean±s.e.m.

To test whether EZH2 may affect *Tbx1* gene expression in two of the critical tissues in which it has a developmental function, we used *in vitro* differentiation protocols to obtain cardiogenic mesoderm and definitive endoderm from mouse ESCs. We targeted *Ezh2* exon 16, encoding part of the methyltransferase domain, using CRISPR/Cas9 technology in E14Tg2A.4 mouse ESCs (Fig. 2A). We selected two clones (1C and 1D) that exhibited homozygous mutation of *Ezh2* and no EZH2 protein expression (Fig. 2A). We subjected these cells (here referred to as *Ezh2*^−/−^ cells) to cardiac mesoderm/heart field progenitor differentiation (Andersen et al., 2018), and to endoderm differentiation (Loh et al., 2014).

During cardiac differentiation, we tested *Gata4*, *Tbx1* and *cTnt* (also known as *Tnnt2*) expression, the first two being markers of cardiac progenitors, and the latter of cardiomyocytes (Fig. 2B). We found that the *Ezh2*^−/−^ cell lines, although positive for these markers, differentiated earlier than the wild-type parental line in two independent experiments. For example, expression of the cardiac troponin gene (*cTnt*), a marker of cardiomyocyte differentiation, appeared at day 4 in mutant cells, but at day 6 in wild-type cells (Fig. 2B). Conversely, *Tbx1* expression appeared later in differentiation and at a lower intensity. qRT-PCR confirmed and quantified these results (Fig. 2C).

**Fig. 3.**
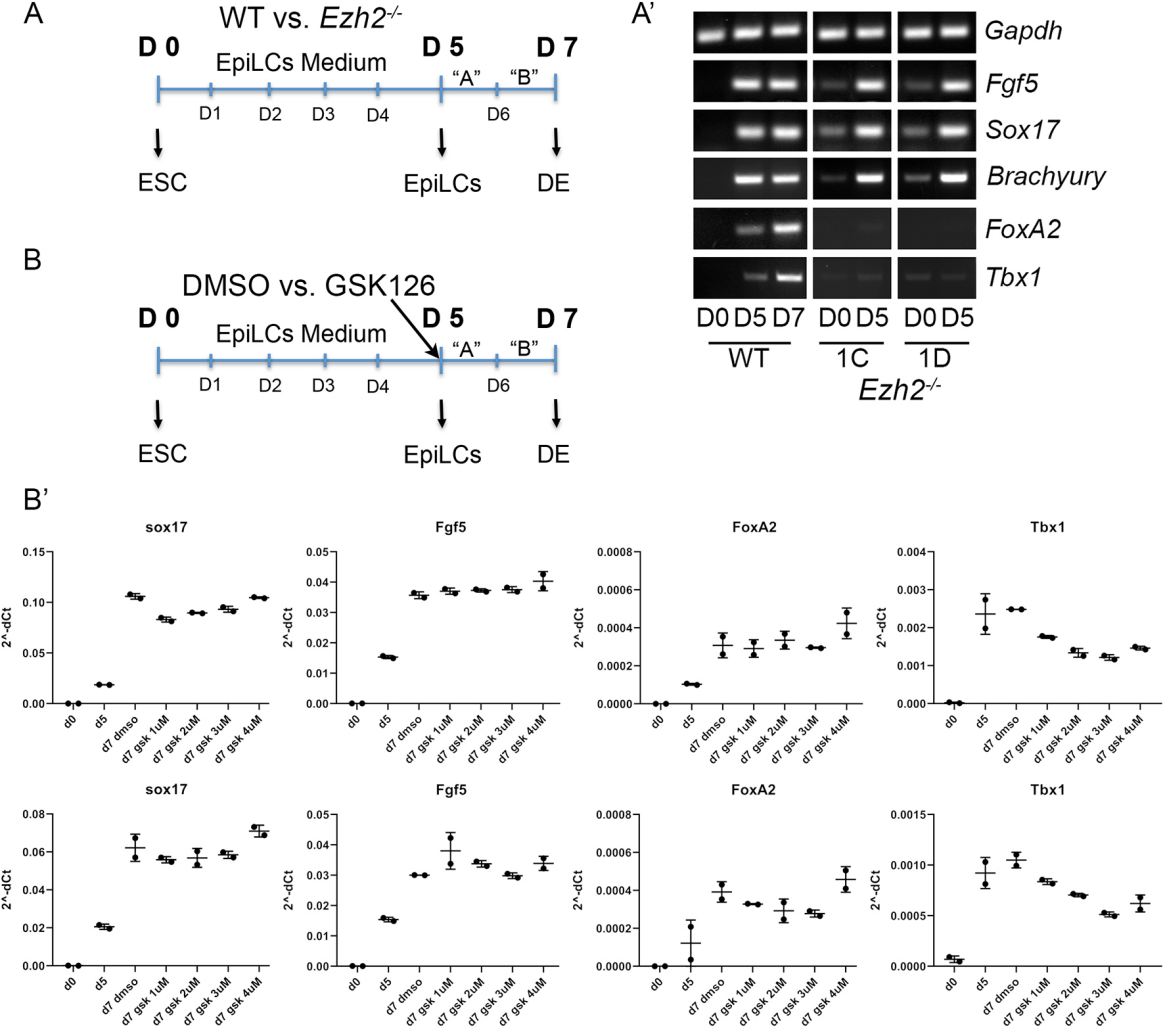
**Loss of EZH2 affects endoderm differentiation.** (A-A′) Definitive endoderm differentiation of *Ezh2*^−/−^ cells using a combination of a protocol to obtain epiblast like cells (EpiLCs) (Fiorenzano et al., 2016) and then definitive endoderm (DE) (Loh et al., 2014). In this set of experiments we were able to obtain epiblast cells from *Ezh2*^−/−^ cells (D5) but could not recover any cells after this time point in three independent experiments. (B-B′) In a separate set of experiments, we have used wild-type (WT) mESCs treated with the EZH2 inhibitor GSK126 at four different concentrations in two independent experiments. (B′) Gene expression measured by qRT-PCR did not show any visible effect of GSK126, except for *Tbx1*, which was downregulated compared to DMSO-treated (control) cells. The two rows of graphs refer to two independent differentiation experiments, each with a technical replicate. Data are mean±s.d.

Endodermal differentiation provided a substantially different picture. Because the protocol was designed for human ESCs, we first induced epiblast differentiation and then proceeded with endodermal differentiation. In the schematic shown in Fig. 3A, time D0 to D5 refer to days of epiblast differentiation, whereas D5 to D7 refer to days of endodermal differentiation (definitive endoderm). As shown in the marker expression panel (Fig. 3A′), at D5, all cell lines robustly expressed the epiblast markers *Fgf5*, *Sox17* and *Brachyury* (*T*); and at D7, wild-type cells expressed the endodermal marker *FoxA2* and also *Tbx1*, which is strongly expressed in the pharyngeal endoderm. However, we could not recover mutant cells at D7 in three independent differentiation experiments. In fact, virtually all cultured mutant cells died between D5 and D6. Thus, with this protocol, the complete loss of EZH2 in these cells prevented endodermal differentiation, expansion or survival. To gain additional information, we inhibited the enzymatic activity of EZH2 using GSK126. We added GSK126 during the endoderm differentiation segment of the protocol (days D5 to D7) at four different concentrations (1 to 4 µM) (Fig. 3B). Under these conditions, we did not observe the culture failures observed with the *Ezh2*^−/−^ cells and were able to harvest RNA for gene expression assays using qRT-PCR. Results showed that GSK126 treatment downregulates the expression of *Tbx1* in two independent differentiation experiments (Fig. 3B′)*.* Of note, the expression of *FoxA2*, suppressed in *Ezh2*^−/−^ epiblast cells, was not affected by GSK126 treatment during endoderm differentiation. Overall, our data indicate that loss of EZH2 is associated with reduced expression of the *Tbx1* gene during cardiac mesoderm and endoderm differentiation.

### Deletion of Ezh2 in the Tbx1 expression domain affects pharyngeal endoderm but not cardiovascular development

**Fig. 4.**
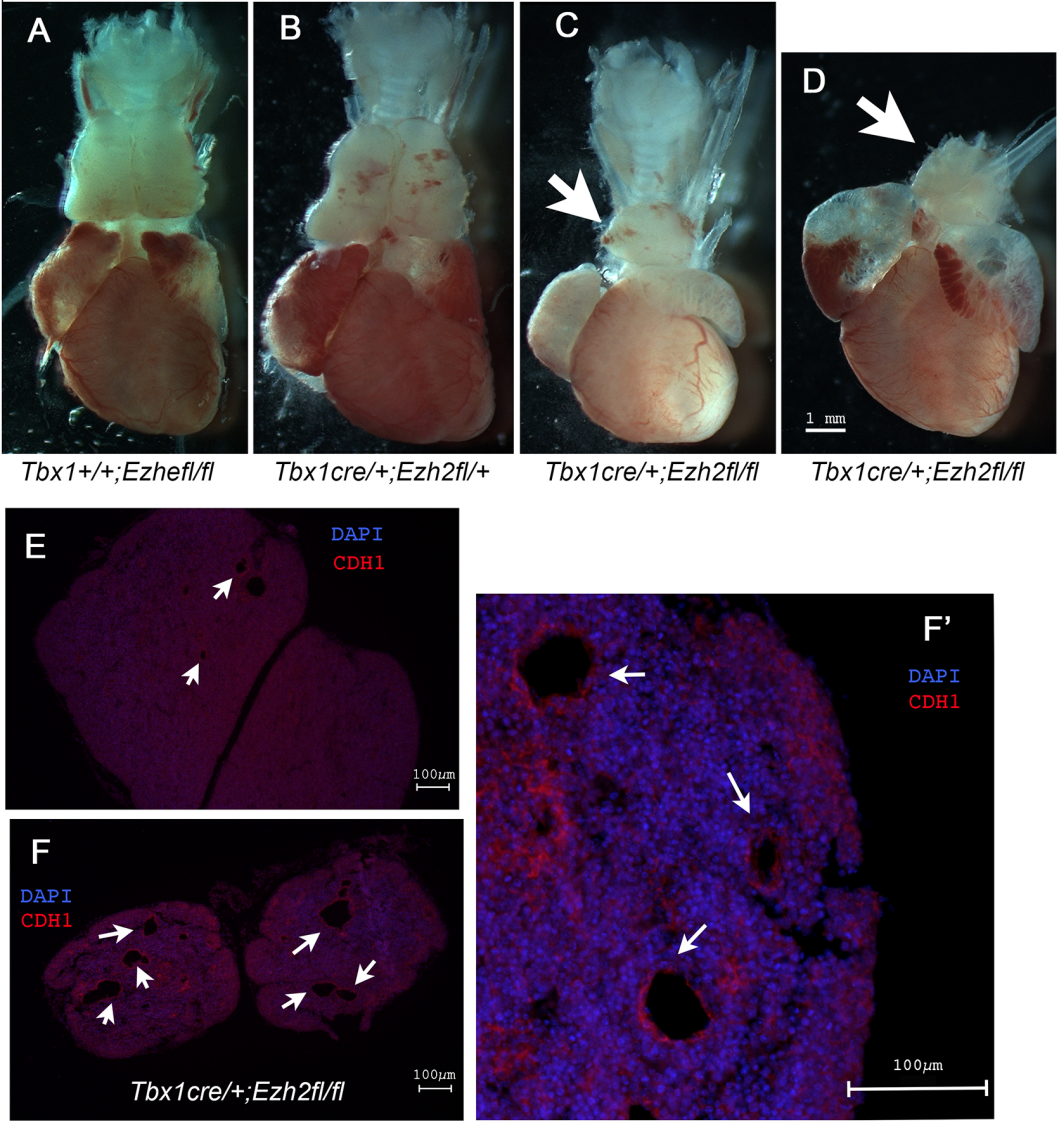
**Loss of *Ezh2* in the *Tbx1* expression domain causes severe thymic hypoplasia in E18.5 embryos.** (A-D) Heart and thymus preparations from E18.5 embryos. Note the small thymus in *Ezh2^cko^* embryos (arrows). (E) Section of an E18.5 control (*Tbx1^Cre/+^;Ezh2^flox/+^*) thymus stained with DAPI (blue for nuclei) and immunostained with an anti E-Cadherin (CDH1) antibody (red). (F) Section of an *Ezh2^cko^* thymus at the same magnification as in E. An adjacent section is shown magnified in F′. Note the large number of CDH1-lined cysts, which are also present, but in a very small number, in control embryos (arrows).

**Fig. 5.**
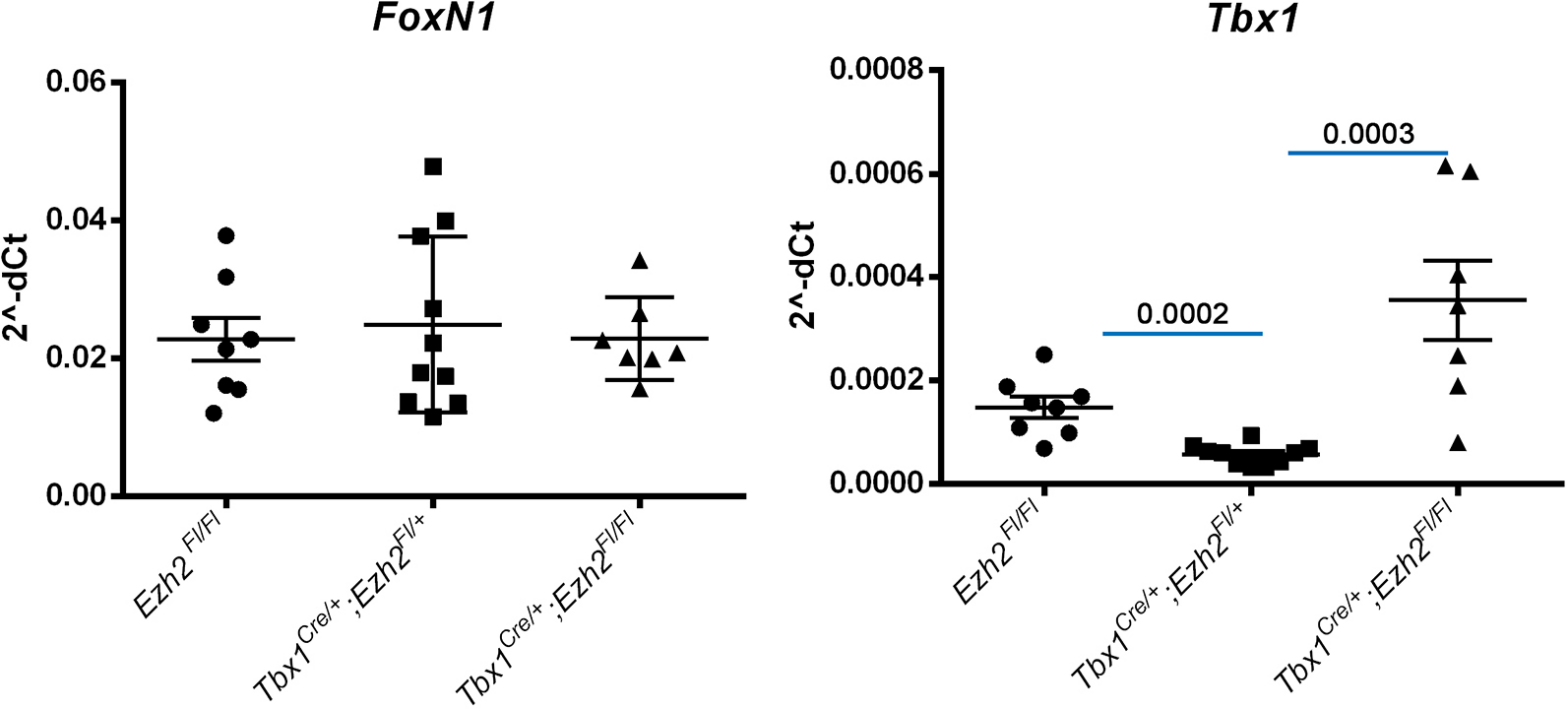
**Normal *FoxN1* but altered *Tbx1* gene expression in E16.5 *Ezh2* mutant thymi.** Quantitative real time PCR of *FoxN1* and *Tbx1* gene expression in thymi of the genotype indicated. *FoxN1* gene expression (left) did not change in any of the genotypes tested. *Tbx1* gene expression (right) was significantly reduced in the double heterozygotes, but increased, and very variable from embryo to embryo, in the *Ezh2^cko^*. Data are mean±s.e.m. Figures above the blue segments indicate *P* values, calculated by an unpaired two-tailed Student's *t*-test using GraphPad software.

Next, we tested whether *Ezh2* may modify the *Tbx1* mutant phenotype. We tested the *Tbx1* haploinsufficiency phenotype and used three different strategies: (1) crosses with germ line mutants (*Tbx1*^+/−^ and *Ezh2*^+/−^); (2) conditional mutation of *Ezh2* (*Tbx1^Cre^*^/+^ and *Ezh2^fl^*^/+^); and (3) EZH2 enzymatic inhibition by GSK126 treatment (Fig. S2). The results, summarized in Table 1, showed that none of these approaches significantly modified the fourth pharyngeal arch artery (PAA) phenotype. Next, we homozygously deleted *Ezh2* in the *Tbx1* expression domain and phenotyped the heart at E18.5 and E16.5. In total, we dissected ten hearts from *Tbx1^Cre^*^/+^;*Ezh2^fl^*^/fl^ (*Ezh2^cko^*) and *Tbx1^Cre^*^/+^;*Ezh2^fl^*^/−^ embryos. None of the hearts examined presented with morphological anomalies (Fig. S1B-E). Although we found no cardiovascular defects, we did find that all of the *Ezh2^cko^* embryos (*n*=15) exhibited thymic hypoplasia (Fig. 4A-D), whereas the other genotypes (including *Tbx1^Cre^*^/+^ and *Tbx1^Cre^*^/+^;*Ezh2^fl^*^/+^) had thymi of normal size. Some of the thymi were sectioned and subjected to immunofluorescence. In mutant embryos, they appeared to be irregular in shape and had many cysts lined by E-cadherin^+^ cells (arrows in Fig. 4E-F′). Cysts of this type were also found in otherwise normal thymi in this strain of mice (arrows in Fig. 4E), but they were uncommon. We then collected another set of embryos at E16.5, extracted RNA from dissected thymi, and processed it for qRT-PCR analysis. We found *FoxN1* gene expression to be similar in control, *Tbx1^Cre^*^/+^;*Ezh2^fl^*^/+^ and *Ezh2^cko^* embryos (Fig. 5, left panel), suggesting that the thymic phenotype was unlikely to derive from the loss of this critical transcription factor (Blackburn et al., 1996; Gordon et al., 2001). *Tbx1* gene expression was very low in control (*Ezh2^fl/fl^*) thymi, at least two orders of magnitude lower than *FoxN1* expression. In *Tbx1^Cre^*^/+^; *Ezh2^fl^*^/+^ thymi, *Tbx1* expression was even lower, consistent with the reduced *Tbx1* gene dosage. In *Ezh2^cko^* thymi, *Tbx1* gene expression was very variable between samples, with three out of seven embryos exhibiting similar levels to controls, whereas the other four embryos expressed higher levels (Fig. 5, right panel). The reasons for this variable *Tbx1* expression in *Ezh2^cko^* thymi is unknown, but it is unlikely to be due to the loss of a suppressive role of EZH2 because this would have increased expression, or at least not reduced it, in *Tbx1^Cre^*^/+^; *Ezh2^fl^*^/+^ thymi. The profound dysmorphogenesis of *Ezh2^cko^* thymi is likely to be associated with an altered contribution of the different cell types that normally make up this organ, namely cells derived from endoderm, neural crest and mesoderm, the latter including thymocytes. Thus, *Ezh2^cko^* thymi might include a higher proportion of cells that express low levels of *Tbx1*, which is variable from sample to sample. Immunofluorescence failed to detect any TBX1^+^ cells in the thymus of any of the genotypes tested, probably because its expression was below the detection sensitivity of this method.

**Table 1.**
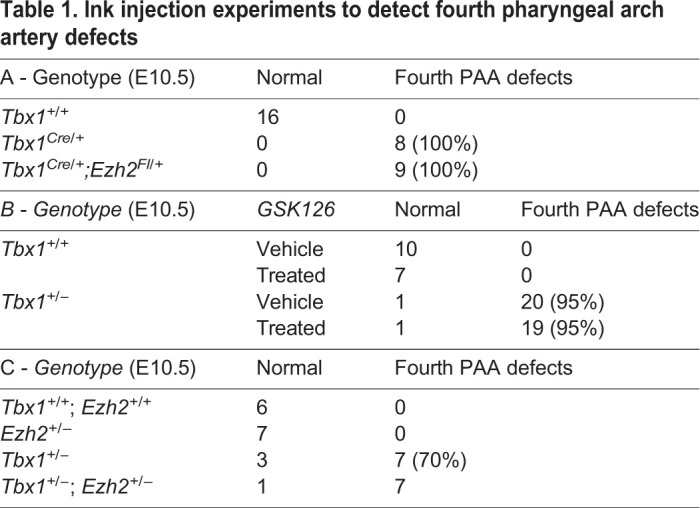
**Ink injection experiments to detect fourth pharyngeal arch artery defects**

**Fig. 6.**
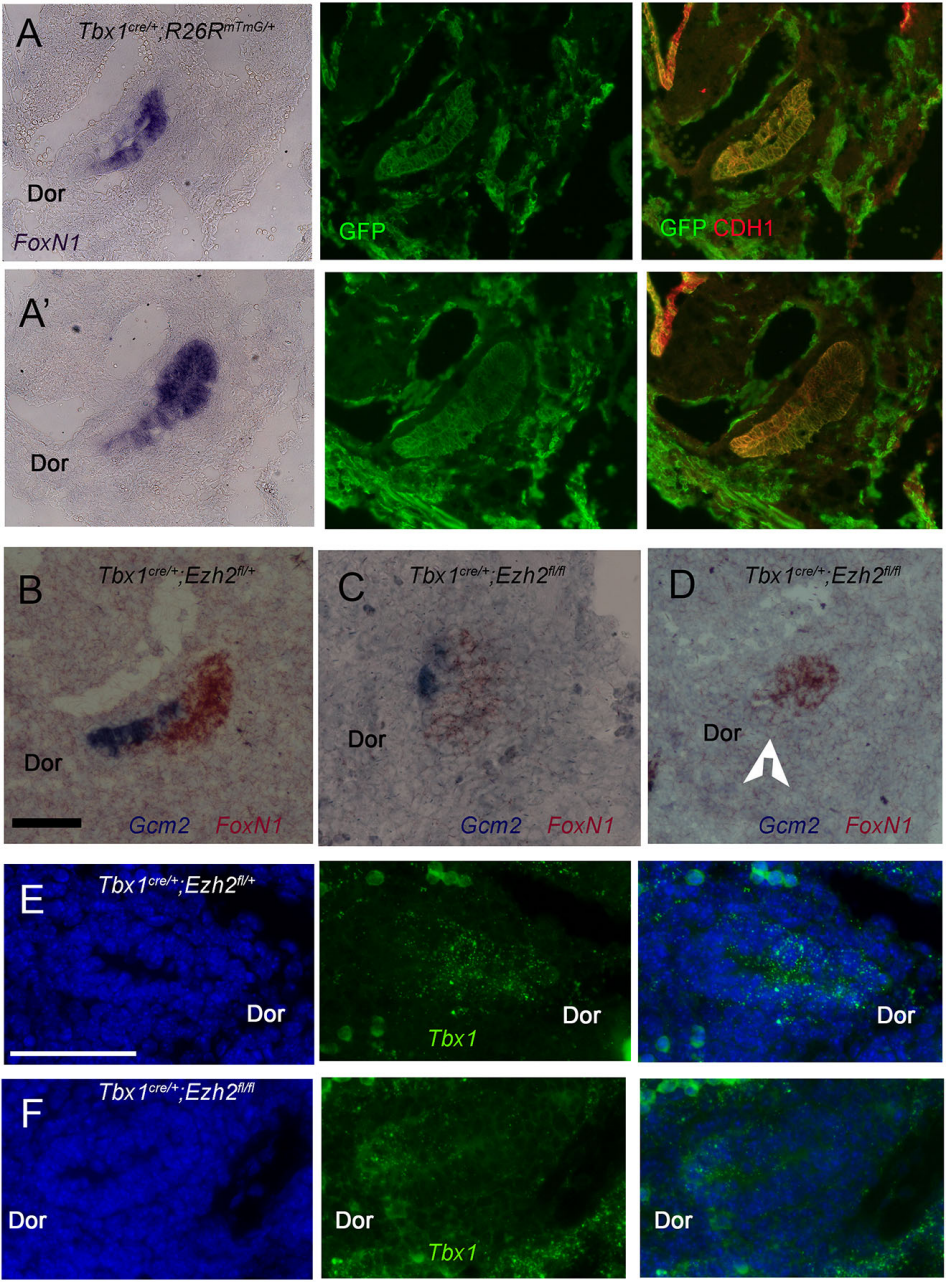
**Third pharyngeal pouch anomalies in *Ezh2^cko^* embryos at E11.5.** (A,A′) Sagittal sections from the same pouch at two different lateral levels stained with a *FoxN1* probe by *in situ* hybridization (left panels). The same sections were then stained with anti GFP (green) and anti CDH1 (red) antibodies. *FoxN1* stains the ventral domain of the pouch, whereas GFP, indicating the recombination of the *Rosa^mT^*^-*mG*^ allele driven by *Tbx1^cre^*^/+^, stains the entire pouch (centre panels), as does CDH1 immunostaining (right panels). (B-D) Two-colour *in situ* hybridization of *Gcm2* (blue) and *FoxN1* (brown) in control (*Tbx1^Cre/+^;Ezh2^flox/+^*) (B) and in two *Ezh2^cko^* embryos (C,D). C shows the only *Ezh2^cko^* pouch in which we could see cells stained with *Gcm2*. In the other embryos no *Gcm2* signal was detected, as in D. The arrowhead indicates a *FoxN1*^*−*^ and *Gcm2*^*−*^ region. (E,F) RNAscope *in situ* hybridization using a *Tbx1* probe (green). Cell nuclei are stained with DAPI (blue). (E) Control embryo (*Tbx1^cre^*^/+^;*Ezh2^flox^*^/+^). (F) *Ezh2^cko^* embryo, which shows a barely detectable signal. Dor, dorsal side of pouch. In all sections anterior is up, posterior is down. Scale bars: 100 µm.

In the mouse, the thymus and parathyroid primordia are derived from adjacent but distinct domains of the third pharyngeal pouch (PP) endoderm, and they express *FoxN1* and *Gcm2*, respectively. *Tbx1* is expressed in the early endoderm that gives rise to both primordia but it is later turned off in the thymic domain (Bain et al., 2016; Huynh et al., 2007). Thus, *Tbx1Cre*-induced recombination, as visualized by the reporter allele *Rosa^mT−mG^*, spans the entire pouch at E11.5, when the domains are clearly distinct (Fig. 6A-A′). In *Ezh2^cko^* embryos, the third PP appeared to be smaller and rounder than in controls (Fig. 6B-D), *FoxN1* was readily detectable but *Gcm2* expression was not, with one exception where we detected 1-2 positive cells (Fig. 6C,D). *Tbx1* expression, revealed by RNAscope, was barely detectable (Fig. 6F), consistent with reduced expression after GSK126 treatment observed in endoderm *in vitro*. Immunofluorescence with an antibody against TBX1 revealed a very low signal in the third PP of *Tbx1^Cre^*^/+^;*Ezh2^fl^*^/+^ embryos, but was undetectable in the third PP of *Ezh2^cko^* embryos (arrows in Fig. S3A-C). We found no obvious difference in the intensity of the signal in adjacent mesodermal tissue (arrowheads in Fig. S3). Despite the absence of *Gcm2* expression, we could see the presence of a *FoxN1*^−^ dorsal region of the mutant pouch (Fig. 6D, arrowhead), a region normally associated with *Gcm2* expression. This suggests that the pouch, although smaller, might be correctly patterned, but failed to activate *Gcm2* expression.

### Ezh2 is required for parathyroid formation

**Fig. 7.**
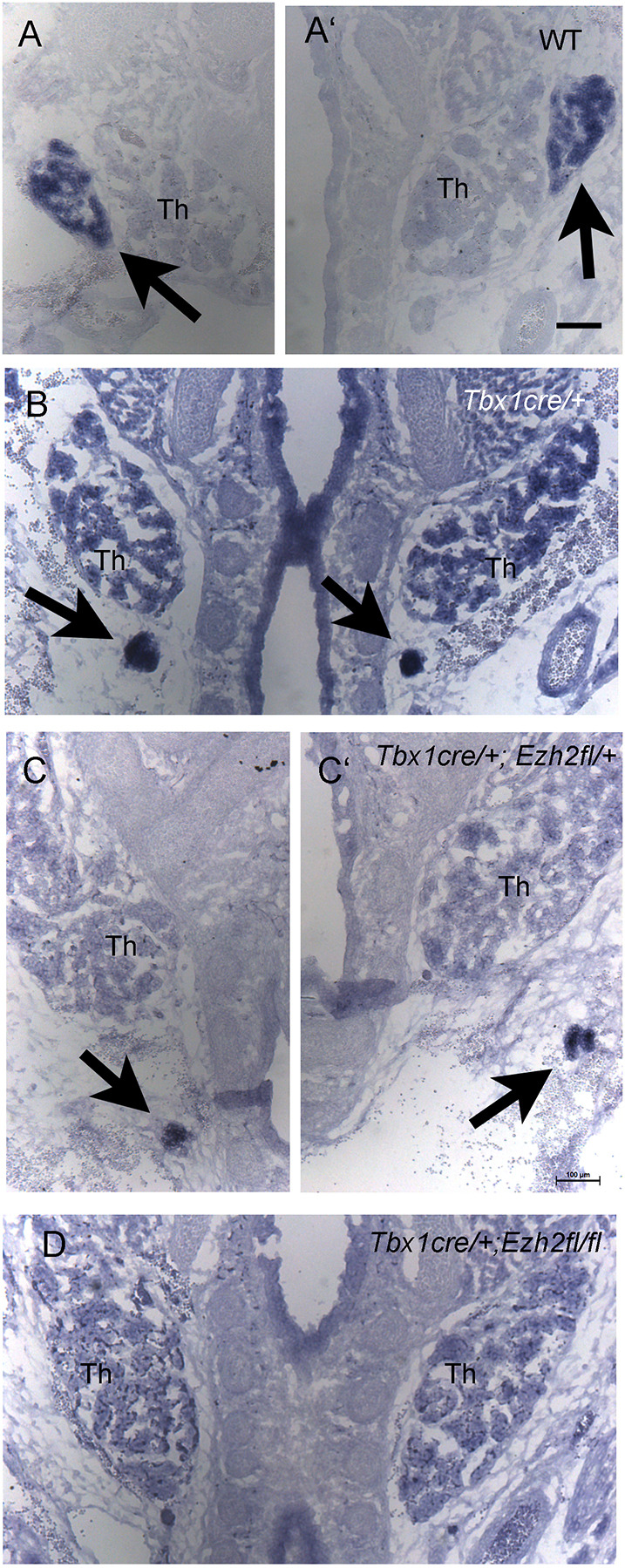
**Parathyroids are undetectable in E16.5 *Ezh2^cko^* embryos.** *In situ* hybridization with *Gcm2* on coronal sections in the mediastinic region. Arrows indicate the parathyroids. (A,A′) Wild-type (WT) embryo (the two half-photographs were taken at different levels to show both lobes). (B) *Tbx1^cre^*^/+^ embryo (heterozygous mutant). Parathyroids are small and ectopic. (C,C′) a *Tbx1^cre^*^/+^;*Ezh2^flox^*^/+^ embryo with small and ectopic parathyroid glands similar to the *Tbx1^cre^*^/+^ genotype. (D) *Ezh2^cko^* mutant that shows no *Gcm2* signal. Th, thyroid lobes. Scale bars: 100 µm.

Because *Gcm2* is required for parathyroid development (Günther et al., 2000; Liu et al., 2007), we tested whether *Ezh2* deletion in the *Tbx1* domain affected parathyroid development. We sectioned E16.5 embryos and examined the neck/mediastinic region by haematoxylin-eosin staining and by *Gcm2 in situ* hybridization. Wild-type embryos exhibited clearly detectable bilateral *Gcm2*-stained parathyroid glands adjacent to the thyroid lobes (Fig. 7A-A′). *Tbx1^Cre^*^/+^ embryos had very small and ectopic parathyroid glands (Fig. 7B), similar to *Tbx1^Cre^*^/+^;*Ezh2^fl^*^/+^ embryos (Fig. 7C), whereas in *Ezh2^cko^* embryos, we could not detect any parathyroid tissue in the entire mediastinic region in coronal and transverse sections. An example is shown in Fig. 7D.

## DISCUSSION

We initiated this work in search of upstream chromatin regulators of the *Tbx1* gene. Published literature suggested that EZH2/PRC2 could be a suppressor of *Tbx1* expression (Collinson et al., 2016; Singarapu et al., 2018), thus a potential target for a compensatory treatment for gene haploinsufficiency. Indeed, ChIP experiments performed on mouse embryos showed that EZH2 localizes to the *Tbx1* gene. However, we did not find any convincing evidence that EZH2 suppresses *Tbx1* gene expression. On the contrary, we found that loss of EZH2, or inhibition of its methyltransferase activity, was associated with reduced expression of the gene in certain contexts.

*Tbx1* is required in a population of mesodermal cells, the SHF, for the development of the cardiac outflow tract and the pharyngeal apparatus (Rana et al., 2014; Xu et al., 2004; Zhang et al., 2006). It is also required in the pharyngeal epithelia, including the pharyngeal endoderm, for the fourth PAA formation and for the development of thymus and parathyroids (Calmont et al., 2009; Hasten and Morrow, 2019; Jackson et al., 2014; Zhang et al., 2005). Thus, we used mouse ESC-based cardiac mesoderm and endoderm differentiation protocols to test the effect of loss of *Ezh2* on *Tbx1* expression. In both systems, loss of EZH2 had a significant impact, although the effect on endoderm differentiation was the most dramatic, as we could not recover any endodermally differentiated cells. However, inhibition of EZH2 enzymatic activity, rather than genetic knockout, allowed us to obtain endodermally differentiated cells for analysis. We observed that suppression of EZH2 reduced the expression of *Tbx1* in cardiac mesoderm and definitive endoderm in cultured cells.

*In vivo* experiments revealed a role of *Ezh2* in the pharyngeal endoderm when ablated in the *Tbx1* expression domain. Indeed, we found that *Ezh2* is required for the development of the third PP, which includes the primordia of the thymus and parathyroids. In E11.5 *Ezh2^cko^* embryos, the third PP was overall hypoplastic, exhibited a *FoxN1*^+^ region, indicative of thymic specification (Blackburn et al., 1996; Gordon et al., 2001), but lacked *Gcm2* expression, which is required for parathyroid development (Günther et al., 2000; Liu et al., 2007; Peissig et al., 2018). Consistent with this, *Ezh2^cko^* embryos exhibited thymic hypoplasia and lacked parathyroids, and in *Tbx1^cre^*^/+^ and *Tbx1^Cre^*^/+^;*Ezh2^fl^*^/+^ embryos the glands were ectopic and hypoplastic. Parathyroid hypoplasia has been reported in *Tbx1* heterozygous mutants (Hasten and Morrow, 2019). Thus, EZH2 is a critical factor in endoderm development and, consequently, it affects the morphogenesis of organs derived from it.

In contrast with a clear effect in the third PP, we found that in a *Tbx1* heterozygous mutant background *Ezh2* loss did not cause cardiac outflow tract defects. This result is consistent with data generated with a different SHF driver, Mef2c-AHF-Cre, and which also showed no outflow tract defects (Delgado-Olguín et al., 2012). Thus, all data considered, *Ezh2* appears to be dispensable for SHF-dependent outflow tract development. These findings do not completely exclude a role of PRC2 in the SHF because loss of *Ezh2* might be compensated by *Ezh1*.

It has been reported that the conditional deletion of *Eed*, encoding another PRC2 component, in the *FoxN1* expression domain causes postnatal thymic hypoplasia and dysfunction (Singarapu et al., 2018). The phenotype was partly attributed to upregulation of the *Tbx1* gene and suppression of the *FoxN1* gene. Our data are in apparent contrast to those results because we found no evidence that *Ezh2* may suppress *Tbx1* expression, although we did see variable expression in mutant thymi, nor did we see *FoxN1* suppression. However, the *Tbx1^Cre^* driver is activated much earlier than the *Foxn1^Cre^* driver, and the loss of the *Ezh2* gene may be partially compensated by *Ezh1*.

In summary, we found that the *Tbx1* gene is a likely target of EZH2 and that its suppression is associated with reduced expression of *Tbx1* in differentiated mouse ESCs and in embryos, at the third PP endoderm. We also observed loss of *Gcm2* expression. Consistently, we observed severe developmental anomalies of the endodermally derived thymus and parathyroids. The mechanisms by which EZH2 regulates *Tbx1* and *Gcm2* expression may be indirect because PRC2 is known to be a repressor, but there are exceptions (Jiao et al., 2020; Kim et al., 2018).

Interestingly, *EZH2* is activated in human parathyroid malignancies (Cetani et al., 2019) and in hyperparathyroidism (Duan et al., 2015), suggesting that the gene has a major role in the transcriptional program of this gland. Finally, 22q11.2DS patients, who are haploinsufficient for *TBX1*, are often, but not always, affected by hypocalcaemia and parathyroid hypoplasia, and therefore it would be of interest to determine whether variants of the *EZH2* gene affect the penetrance and/or expressivity of this clinically relevant phenotype.

## MATERIALS AND METHODS

### Mouse lines

We used the following mouse lines; *Tbx1*^Cre/+^ (Huynh et al., 2007); *Tbx1*^+/−^ (Lindsay et al., 2001), *Ezh2^fl/fl^* (*Ezh2^tm1TaraGrea!^*) (Su et al., 2003); Mef2c-AHFCre (Verzi et al., 2005); and *Rosa^mT^*^-*mG*^, a Cre reporter line (Muzumdar et al., 2007). All lines were maintained in a congenic C57/Bl6N background. To generate an *Ezh2* null allele, Mef2c-AHFCre females, which express Cre in the germline (Ehlers et al., 2014) were crossed with *Ezh2^fl/+^* mice. *Ezh2*^+/−^ progeny were crossed with wild-type mice and we used the second generation of *Ezh2*^+/−^ mice to exclude mosaicism. All animals were genotyped according to the original reports. To evaluate the fourth PAA phenotype, we injected India ink into the heart of E10.5 embryos. Subsequently, embryos were fixed in 4% paraformaldehyde (PFA), dehydrated and clarified with 1:1 benzyl-alcohol benzyl-benzoate. To inhibit EZH2 enzymatic activity, we crossed *Tbx1*^+/−^ with wild-type mice, and pregnant females were injected with 50 mg/kg of GSK126 (Selleck Chemicals), at E7.5, E8.5 and E9.5. GSK126 was dissolved in 20% Captisol (CyDex Pharmaceuticals), adjusted to pH 4-4.5 with 1 N acetic acid. Equivalent amounts of diluted Captisol were injected in control mice.

Mouse studies were performed under the auspices of animal protocol 257/2015-PR (licensed to the AB lab) reviewed, according to Italian law, by the Italian Istituto Superiore di Sanità and approved by the Italian Ministero della Salute. The laboratory applies the ‘3Rs’ principles to minimize the use of animals and to limit or eliminate suffering.

### Cell cultures

E14Tg2A.4 feeder-free mouse ESCs (strain 129/Ola, BayGenomics) were cultured in feeders-free conditions in Glasgow's minimum essential medium (Sigma-Aldrich, G5154) supplemented with 15% ESC-screened fetal bovine serum (FBS) (US Euroclone, CHA30070L), 0.1 mM non-essential amino acids, 0.1 mM β-mercaptoethanol, 0.1 mM L-glutamine, 0.1 mM sodium pyruvate and 10^3^ U/ml ESGRO leukaemia inhibitory factor (Millipore, ESG1107). Cardiac differentiation was performed according to Andersen et al. (2018). The organoids were collected every 2 days to monitor cardiac differentiation. We performed epiblast differentiation according to a published protocol (Fiorenzano et al., 2016). Endoderm differentiation was performed using a commercial kit (PSC Definitive Endoderm Induction Kit, Gibco, A3062601) based on published data (Loh et al., 2014). For experiments with the inhibitor GSK126, the inhibitor was dissolved in DMSO (Sigma-Aldrich, 1 to 4 µM final concentration in the medium) and added to the culture at day 5 (beginning of endoderm differentiation). The same volume of DMSO was added to control cultures.

CRISPR/Cas9-mediated targeting of the *Ezh2* gene was performed using a guide (g)RNA sequence (Fig. 2) that was selected, using the tool crispr.mit.edu, to have the lowest off target probability. The sequence was cloned into a plasmid (Thermo Fisher Scientific, A21174) encoding the single gRNA and the Cas9 protein, and an orange fluorescent protein. The plasmid was transfected into mouse ESCs using X-tremeGENE, along with an oligonucleotide as a homologous recombination template. Transfected cells were FAC-purified and plated at clonal density. Forty-three clones were picked, expanded and screened by PCR to identify clones that had undergone homologous recombination. Targeted clones were sequenced confirmed and then tested by western blot to verify the loss of the EZH2 protein. All cell cultures used in this work were regularly tested to exclude mycoplasma contamination.

### Chromatin immunoprecipitation

E9.5 embryos were collected and fixed in 1% formaldehyde for 12 min, stored at −80°C and, after chromatin extraction, we immunoprecipitated 6 µg of chromatin. ChIP experiments were carried out as described previously (Lania et al., 2016).

### Histone extraction

Histone extraction was performed as previously described (Gordon et al., 2001; Shechter et al., 2007). Briefly, embryos were homogenized with hypotonic lysis buffer [10 mM Tris-Cl (pH 8.0), 1 mM KCl, 1.5 mM MgCl_2_, 1 mM DTT and protease inhibitors] and incubated for 30 min. To extract histones, the nuclei were collected by centrifugation at 10,000 ***g*** and resuspended in acid extraction buffer (0.4 N H_2_SO). The histone preparation was concentrated by precipitation with trichloroacetic acid and resuspended in deionized water for western blot analysis. Antibodies are listed in Table 2.

**Table 2.**
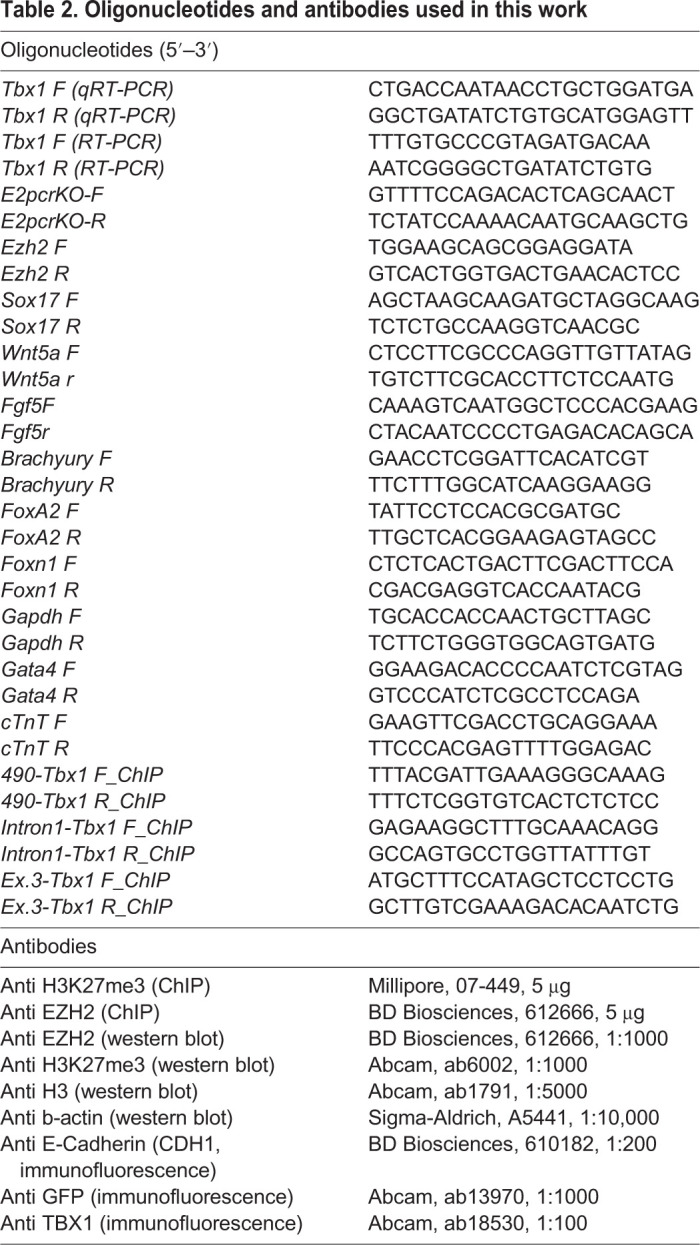
**Oligonucleotides and antibodies used in this work**

### Immunofluorescence

For immunofluorescence analysis, tissues were embedded in paraffin and cut into 10 µm sections. Sections were rehydrated and briefly microwaved to boiling point in 10 mM sodium citrate (pH 6.0). Slides were then incubated with the primary antibody overnight in blocking solution (0.5% milk, 10% FBS and 1% bovine serum albumin in H_2_O). Signal was revealed with fluorochrome-conjugated secondary antibodies (Table 2).

To perform the immunofluorescence analysis following *in situ* hybridisation on E11.5 embryos, at the end of the *in situ* hybridisation protocol, slides were washed in PBS-0.5% Triton X-100 and incubated with primary antibody. Signal was revealed as described above.

### RNAscope *in situ* hybridization

RNAscope experiments were performed according to the manufacturer's instructions. Embryos were fixed in 4% PFA at 4°C, then dehydrated using a standard ethanol series and embedded in paraffin. Selected sections of 6 µm were hybridized with RNAscope Probe Mm-Tbx1 (ACD, 481911, probe C1).

### *In situ* hybridization

For *in situ* hybridization, antisense RNA probes were labelled using a digoxigenin RNA and fluorescein RNA labelling kit (Roche). The probes used were for *Gcm2* (Addgene, 41031) and *FoxN1* (Addgene 41032) (Gordon et al., 2001). E11.5 mouse embryos were cryoprotected by serial dilution of sucrose/1× PBS (10%, 20% and 30% sucrose) at 4°C and then incubated for 2 h at 4°C in 50:50 v/v 30% sucrose/PBS 1× optimal cutting temperature (OCT) compound, then embedded in OCT compound and cut into 10 µm sections. To perform the *in situ* hybridization at E16.5, embryos were embedded in OCT compound as described above.

### RNA extraction and qRT-PCR

Total RNA was isolated from P19Cl6, mouse ESCs and embryos using TRIzol. RNA (0.2-1 µg) was retrotranscribed using the High-Capacity cDNA Reverse Transcription kit (Applied Biosystems, Foster City, CA, USA) and diluted tenfold with RNase-free H_2_O. Gene expression was quantified by qRT-PCR using 2 µl of the reverse transcription reaction and the Power SYBR Green PCR Master Mix (Applied Biosystems), and normalized relative to *Gapdh* expression. Statistical evaluation was performed using a two-tailed Student's *t*-test. Primers used are listed in Table 2.

## Supplementary Material

Supplementary information
