# Supplementary material for: EZH2 is required for parathyroid and thymic development through differentiation of the third pharyngeal pouch endoderm
Source: Dis Model Mech. 2021 Mar 12;14(3):dmm046789. doi: 10.1242/dmm.046789 (PMC7969367; doi:10.1242/dmm.046789)
Supplement: Supplementary information [file dmm-14-046789-s1.pdf]

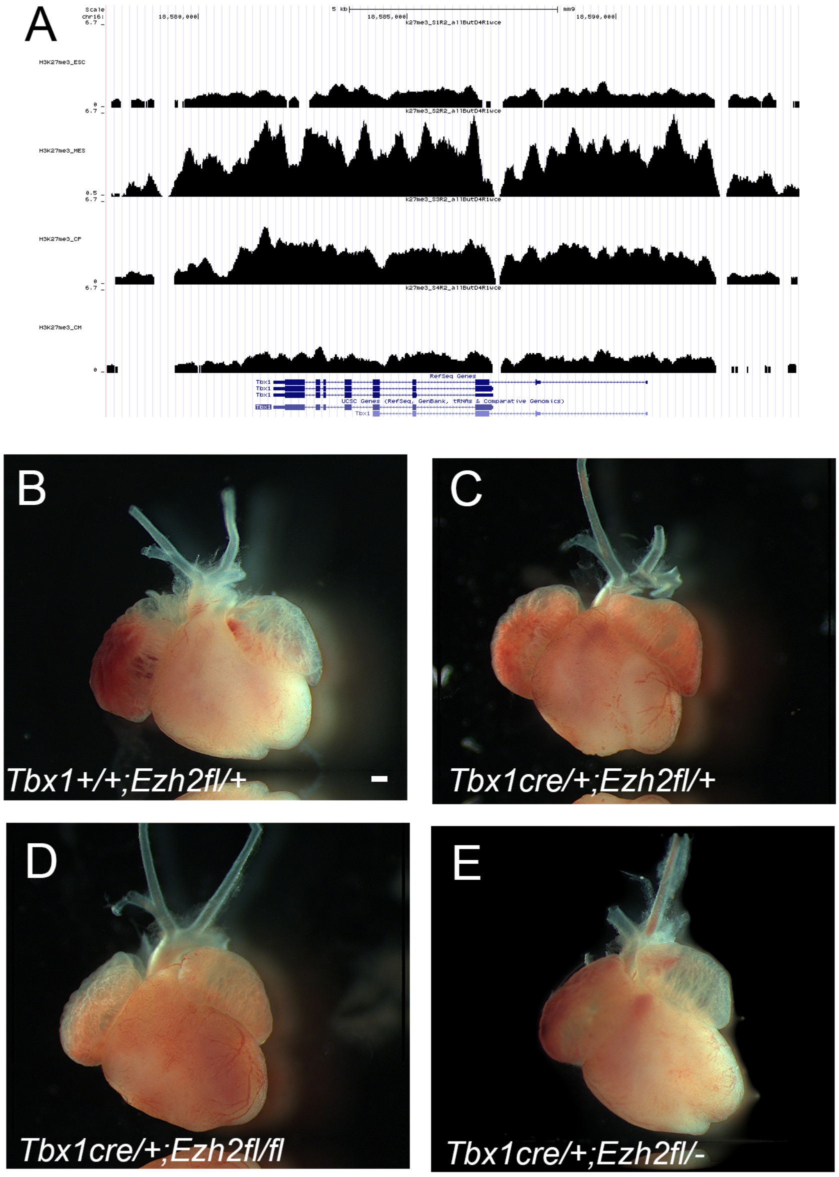

Figure S1

A) Screen shot of genome browser showing H3K27me3 enrichment of the Tbx1 gene region in mESCs undifferentiated (top row), differentiated into mesoderm (second row from top), differentiated into cardiac progenitors (third row), and cardiomyocytes (bottom row). Data downloaded from reference (Wamstad et al., 2012). Graphs are shown with the same scale, note diffuse enrichment over the gene body. Tbx1 is expressed in the mesoderm and cardiac progenitor stages only.

B-E) Isolated hearts from E16.5 embryos with the indicated genotypes. All hearts are morphologically normal and were also sectioned to exclude internal anomalies. Scale bar indicates 200μm.

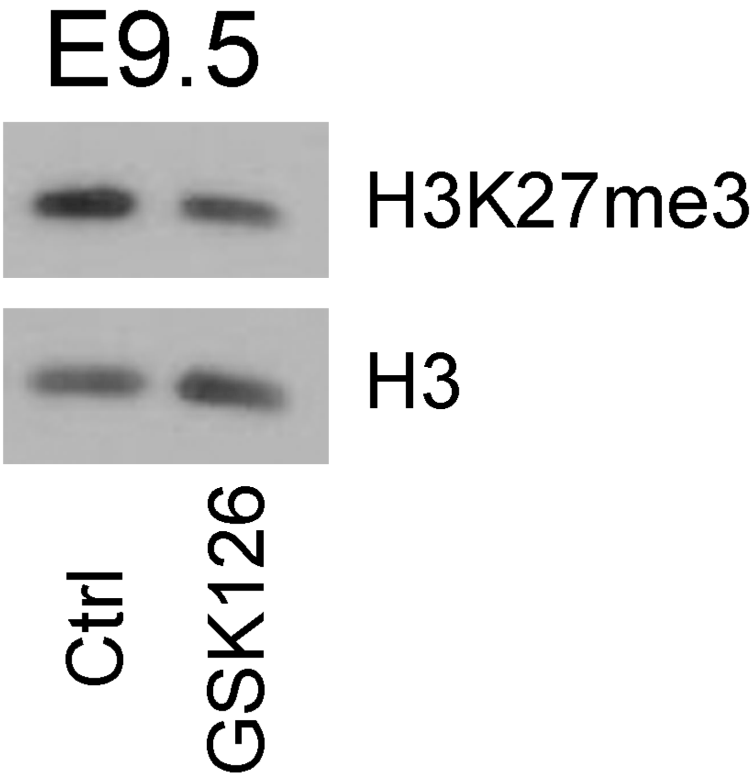

Figure S2
Western blot of histones extracted from E9.5 embryos (WT) treated with vehicle (Ctrl) or GSK126, an inhibitor of EZH2 enzymatic activity. Each lane corresponds to an individual embryo. The experiment was repeated with two sets of embryos and technical triplicates.

Disease Models & Mechanisms • Supplementary information

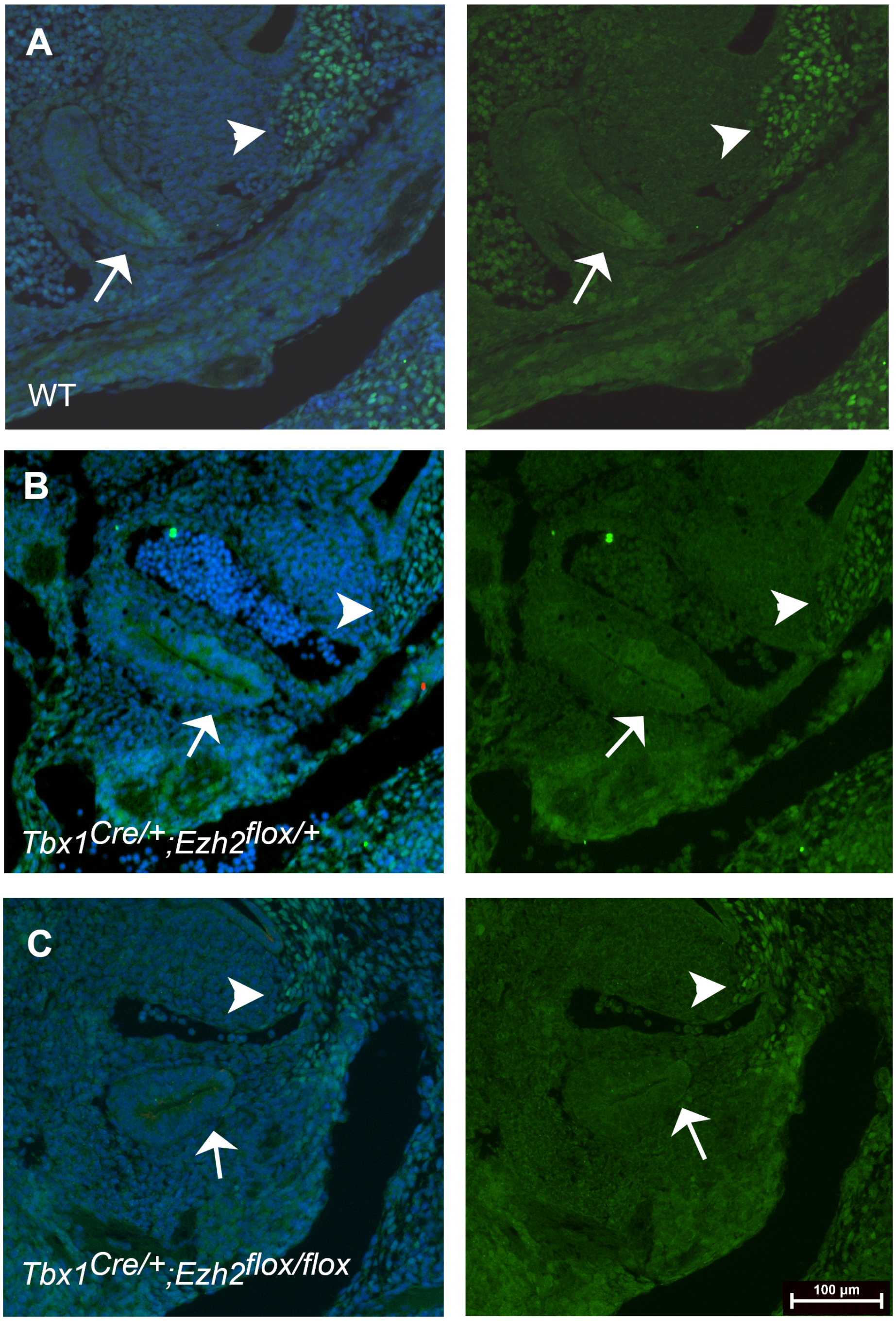

Figure S3

TBX1 immunofluorescence experiments of sagittal sections from E11.5 embryos. (A) Wild type (WT). (B) Tbx1Cre/+;Ezh2flox/+. (C) Tbx1Cre/+;Ezh2flox/flox. Panels on the left are merged images (blue for DAPI and green for TBX1 immunostaining). Panels on the right show only the TBX1 signal. Arrows indicate the third pharyngeal pouch. Arrowheads indicate a TBX1-expressing cell population of the pharyngeal mesoderm that shows a similar intensity regardless of the genotype. No signal could be detected on the third pharyngeal pouch of the mutant in C, Scale bar: 100μm. All images were collected at the same magnification.
